# Case of Embryulcia

**Published:** 1811-05

**Authors:** H. G. Clough

**Affiliations:** Berners-street


### *To the Editors of the Medical and Physical Journal.*

### *Case of Embryulcia.*

Gentlemen,

As the recording of cases has not been much attended to since the time of Smellie's publication, the history of particular occurrences in the art of midwifery, comparatively with those in the other branches of medical science being few in number, and as it is indisputable that the publication of select cases tends to add to our stock of knowledge, I have transmitted to you the following instance of the *operation of embryulcia;* and I request, if you you approve of it, to see it inserted in your extensively circulated Journal.

The process of natural labour is, generally speaking, uniform; but at the same time, we well know, is always attended with pain, and oftentimes with danger. After the operation of embryulcia has been performed, in many cases of very difficult parturition, labour is also frequently retarded, and not without considerable danger to the patient, by reason of the extreme deformity of the pelvis, or disproportion of its cavity to the volume of the cranium, or the wrong position of its base; from which latter circumstance it cannot readily be extracted in the long diameter of the superior aperture of the pelvis, by the blunt hook or crotchet after this operation. Perplexing as these cases are thus rendered, this operation is still preferable to the cæsarean section, where the child is not sacrificed for the preservation of the mother, but where the mother is, in fact, devoted to the child: or even to that of the section of the symphisis pubis, where both mother and child are involved in the same impending danger.

Mary Osborne, the wife of a butcher, No. 6, Phœnix-street, near the Seven Dials, about twenty-four years of age, in her second pregnancy, was admitted as a lying-in patient at the St. Mary-le-bone General Dispensary, in November last; the child of which she was delivered in the former case was born dead, though large, without the use of instruments, and she had passed a tolerable good share of health since that period to the time of her delivery in the present case. She was taken in labour on Tuesday morning the 19th of February, with the usual symptoms accompanying its accession, and was attended by one of the midwives belonging to the charity, during the course of that day; the child still remaining very high up, and finding it impossible to deliver her, she sent for me on Wednesday morning following at nine. Being unable to attend by reason of the indisposition of part of my family, and having been up many preceding nights, I requested my superintending pupil, Mr. Andrews, a gentleman of very promising abilities, to attend for me. He remained with her the whole of that day and night, without any prospect of success. On the morning following, the 21st, he returned much about the same hour, and requested me to accompany him with instruments, observing, it was impossible the delivery could be otherwise accomplished, as the cranium was very large, and resting on the superior aperture of the pelvis, in the short diameter. Accordingly I went, and passing my examination, I made the following deductions on the state of the case: that as the cranium was very large, and completely ossified at the sutures and fontanelles, and resting as it did with the occiput on the base of the sacrum and face over the pubis; a considerable considerable angle being formed at the base, not admitting more than two fingers, or about two inches distance from sacrum to pubis; an adventitious tumour also lining nearly one half of the vagina, which as it happened did not impede either the progress, or any of my future operations in the completion of the labour, I considered it of little importance, but from the above circumstance, and the poor woman having been for some time in very strong convulsions, which still continuing, I determined on the propriety of the operation in the first instance, rather than submit her to the long sufferance of a tedious and difficult case, to the risk of life. I formed my aperture at that part of the cranium, near the posterior fontanelle, on the left parietal bone, which I penetrated with considerable difficulty beyond the knobs of the instrument, on the opening of which I was obliged to get assistance, the consolidation of the bones being so great. I formed another aperture near the pubis with the same difficulty, and after several hours labour, and the contents evacuated, I extracted about four pieces of parietal and frontal bones, but could not fix the crotchet in the great foramen, or, in short, in any part, to get it in the proper direction of the long diameter of the pelvis. Ultimately, about three o'clock in the afternoon, I got three fingers of my right hand introduced laterally between pubis and sacrum, and through the integuments of the cranium up to its base, the middle one of which I passed into the foramen magnum occipitalis, and the other two along the clenoid processes of the ethmoid bone, and giving it a turn, succeeded in placing it in the right direction. Having yet a difficulty to overcome in getting the base of the cranium edge ways, and not being able to fix the crotchet on the internal part, I obtained possession of a portion of the integuments, and fixing the integuments on the external part of the occipital bone, passed it through the superior aperture, and after much exertion through the external orifice. I had nearly the same trouble in getting the shoulders through in the right direction, they were so very large. So far the labour was completed. Had the labour continued much longer, I must inevitably have lost my patient. The placenta was brought away easily in the usual manner, in about half an hour. A few days afterwards she was seized with peritoneal inflammation, which soon subsided by proper treatment, and is now perfectly recovered from the parturient state.

I am, Gentlemen,

Your humble Servant,

H. G. CLOUGH, M. D.

