# Corrigenda

**Published:** 1811-06

**Authors:** 


### *CORRIGENDA.*

In the Case of Embryulcia, by Dr. Clough, at p. 389, line 25, for *Ethmoid*, read *Sphenoid*; and line 30, for *Integuments*, read *Instruments*.

